# AtKATANIN1 Modulates Microtubule Depolymerization and Reorganization in Response to Salt Stress in *Arabidopsis*

**DOI:** 10.3390/ijms21010138

**Published:** 2019-12-24

**Authors:** Jie Yang, Bang An, Hongli Luo, Chaozu He, Qiannan Wang

**Affiliations:** Hainan Key Laboratory for Sustainable Utilization of Tropical Bioresource, College of Tropical Crops, Hainan University, Haikou 570228, China; 17071010210020@hainanu.edu.cn (J.Y.); anbang@hainu.edu.cn (B.A.); hlluo@hainu.edu.cn (H.L.); czhe@hainu.edu.cn (C.H.)

**Keywords:** *Arabidopsis*, KATANIN1, microtubule, organization, dynamics, salt stress

## Abstract

The microtubule cytoskeleton is a dynamic system that plays vital roles in fundamental cellular processes and in responses to environmental stumili. Salt stress induced depolymerization and reorganization of microtubules are believed to function in the promotion of survival in *Arabidopsis*. Microtubule-severing enzyme ATKATANIN1 (AtKTN1) is recognized as a MAP that help to maintain organized microtubule structure. To date, whether AtKTN1 is involved in response to salt stress in *Arabidopsis* remains unknown. Here, our phenotypic analysis showed that the overexpression of *AtKTN1* decreased tolerance to salt stress, whereas the knock-out of *AtKTN1* increased salt tolerance in the early stage but decreased salt tolerance in the later stage. Microscopic analysis revealed that microtubule organization and dynamics are distorted in both overexpression and mutant cells which, in turn, resulted in an abnormal disassembly and reorganization under salt stress. Moreover, qRT analysis revealed that stress-responsive genes were down-regulated in overexpression and mutant cells compared to WT cells under salt stress. Taken together, our results indicated roles of AtKTN1 in modulating microtubule organization, salt-stress induced microtubule disruption and recovery, and its involvement in stress-related signaling pathways.

## 1. Introduction

Salinity, one of the major environmental factors leading to poor crop growth and yields, impacts over 6% of the world’s land area [1]. Salt stress in plants induces osmotic, ionic, and secondary stresses, especially oxidative stress [2,3]. Plants rely on various signaling pathways that rebuild cellular osmotic, ionic, and ROS homeostasis, to modulate plant growth and survival under salt stress [4].

Microtubule is an important cytoskeletal system crucial for numerous fundamental cellular processes [5,6,7,8]. Among multiple signaling pathways for the response to stresses, microtubule is also a common downstream target [9,10]. Microtubule array is highly dynamic consequent on rapid microtubule disassembly and assembly, both of which are believed to be necessary for plant cells in response to environment stimuli including salt stress [9,11,12]. There is mounting evidence demonstrating the effects of salt stress on microtubule organization and dynamics. Cortical microtubule array in *Zea mays* root cells reoriented from transverse to parallel pattern to the longitudinal axis under salt stress [13]. The transverse pattern of microtubule organization in tobacco BY-2 cells rearranged to a random structure after being exposed to 150 mM NaCl for 15 min [14]. The stabilization of microtubules resulted in a lower survival rate of *Arabidopsis* seedlings under salt stress, while the disruption of microtubules improved salt tolerance [9]. PLDa1-generated lipid messenger PA interacts with Microtubule-Associated Protein 65-1 (MAP65-1) and contributes to microtubule stabilization, thereby leading to the promotion of salt tolerance [15]. RIC, an effector of Rho-related GTPase from plants (ROPs), negatively modulates microtubule reorganization caused by salt treatment. And ROP2 activity promotion leads to the reduction of RIC1 on microtubule [16]. *Arabidopsis* Histone H2B monoubiquitination (H2Bub1) modulates salt stress-induced microtubule disassembly, and regulates the PTP-MPK3/6 signaling pathway which participates in the modulation of microtubule stabilization [17]. *Oryza sativa* E3 ligase Microtubule-Associated RING finger protein 1 (OsMAR1) negatively regulates the salt-stress tolerance by the interaction and degradation of *Oryza sativa* chymotrypsin protease inhibitor 2 (OCPI2) [18]. In addition, recent study showed that ethylene signaling regulated the expression of microtubule-stabilizing protein WAVEDAMPENED2-LIKE5 (WDL5), contributed to the modulation of microtubule organization and dynamics in *Arabidopsis* under salt stress [19].

It is well known that microtubule organization and dynamics are regulated by Microtubule Associated Proteins (MAPs) in response to internal and external cues [20,21]. Microtubule-severing enzyme katanin was identified as a vital MAP in setting up the organized state of microtubule arrangement [22,23,24,25]. Katanin is consist of two subunits p60 (60-kDa catalytic ATPase) and p80 (80-kDa WD-40 repeat-containing regulatory subunit), and they oligomerize into a ring complex through the conserved AAA+ domain in the presence of ATP in vitro [26,27]. In animal cells, p80 subunits are not required for severing activity, but help katanin complexes with anchoring to centrosomes [28]. The p60 subunit of *Arabidopsis* AtKTN1 is capable of performing microtubule severing activity in vitro [23], and is mainly recruited to microtubule branching nucleation sites and crossovers to trigger severing in vivo [25,29,30,31]. A recent study showed that four *Arabidopsis* KTN80s act redundantly during plant development and function in the targeting of katanin complexes at both crossovers and branching nucleation sites [32]. Loss-of-function mutations of the katanin p60 subunit in both *Arabidopsis* and *Rice* caused more bulged cells and dwarfed plants because of disorganized microtubule arrays [22,24,33,34]. Further, the deletion of AtKTN1 abolished severing activity in vivo [34].

Given the presence of microtubule disassembly and reorganization in salt resistance, and importance of AtKTN1 in controlling microtubule organization and dynamics, we hypothesized that AtKTN1 might be involved in responses to salt stress. In this study, the role of AtKTN1 in salt stress response and the possible mechanism for it were investigated. By observation and comparison of the salt tolerant phenotype of WT (Col-0), *ktn1-4* [35] and *35S::KTN1*, we found knock-out that mutant *ktn1-4* seedlings showed relatively lower sensitivity in the early stage of salt treatment but higher sensitivity in later stages. Meanwhile, the overexpression of *AtKTN1* presented decreased salt tolerance significantly in the whole treatment. To elucidate the underlying mechanism of AtKTN1 in regulating responses to salt stress, further analysis of their organization and dynamics were performed, and the results suggested that AtKTN1 played important roles in modulating salt stress-induced microtubule disassembly and reorganization by manipulating microtubule severing. The downregulation of stress induced genes in *ktn1-4* and *35S::KTN1* seedlings suggested that AtKTN1 was also involved in stress related signaling pathways.

## 2. Results

### 2.1. Depletion of AtKTN1 Improved Salt Tolerance in the Early Stage of Salt Treatment but Reduced in the Later Stage. Overexpression of AtKTN1 Have Decreased Salt Tolerance Significantly

**Figure 1:**
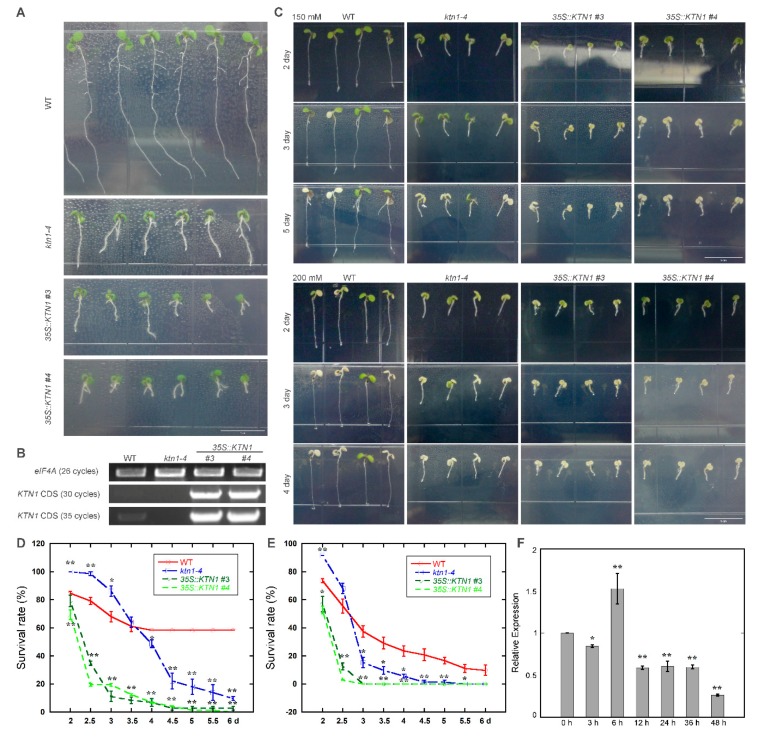
Knock-out of *AtKTN1* improves salt tolerance in the early stage of salt treatment but reduces in the later stage. Overexpression of *AtKTN1* decreases salt tolerance significantly. (**A**) Seven-day-old *Arabidopsis* seedlings WT (Col-0), *ktn1-4* and *35S::KTN1* (#3, #4) grown under lights. Scale bar = 1 cm. (**B**) RT-PCR analysis of the transcriptional levels of *KTN1* in WT (Col-0), *ktn1-4* and *35S::KTN1* (#3, #4) seven-day seedlings. *eIF4A* was used as the control gene. (**C**) Salt sensitivity of WT (Col-0), *ktn1-4* and *35S::KTN1* (#3, #4) seedlings under 150 mM and 200 mM NaCl. Seedlings (four-day-old) of wild type and mutants were transferred from 1/2 MS medium to 1/2 MS medium supplemented without or with 150 mM and 200 mM NaCl. Scale bar = 1 cm. (**D**) Survival rates of WT (Col-0), *ktn1-4* and *35S::KTN1* (#3, #4) seedlings under 150 mM NaCl. (**E**) Survival rates of WT (Col-0), *ktn1-4* and *35S::KTN1* (#3, #4) seedlings under 200 mM NaCl. Surviving seedlings (with green cotyledons) were counted from the second day after transfer. Data shown are mean values SE (error bars) from three independent experiments (n = 48 for each). Asterisks indicate the significance of the mean value differences compared with WT by Student’s *t*-test, * *P* < 0.05,** *P* < 0.01. (**F**) Relative expression levels of *AtKTN1* after treatment with 200 mM NaCl for indicated time period in WT seedlings (four-day-old). Error bars indicate SD. * *P* < 0.05 and ** *P* < 0.01 by a Student’s *t* test.

To identify the function of AtKTN1 in response to salt stress, wild-type (Col-0), T-DNA mutant line *ktn1-4* and overexpression lines *35S::KTN1* (#3 and #4) were used (Figure 1A,B). Four-day-old seedlings grown on 1/2 MS were transferred to 1/2 MS without or supplemented with 150 or 200 mM NaCl for phenotypic analysis. After treatment with 150 mM NaCl for three days, *35S::KTN1* lines showed remarkable salt injury, with nearly all of the cotyledons being discoloured and bleached. In comparison, only a small portion of cotyledons of WT and *ktn1-4* were discoloured and bleached. While after treatment for another two days, the survival rate of *ktn1-4* declined significantly, with most of the cotyledons being discoloured and bleached, and WT still showed relative healthier growth state (Figure 1C). Meanwhile, the survial rate of *ktn1-4* was 18% higher than that of WT during the first three days of treatment, but then decreased sharply to about 10% as the treatment time increase to 6 day (Figure 1D). A similar phenomenon has been observed when treated with NaCl at 200 mM. For *35S::KTN1* seedlings, cotyledons were discoloured and bleached significantly, and the survival rate decreased to 0% after the treatment for three days. Further, *ktn1-4* showed a higher survival rate than WT at the after 2.5 days, and then decreased dramatically to less than 20% after three days (Figure 1E). Salt-sensitive phenotype in soil-grown plants was also analyzed by irrigating four-week-old seedlings with 400 mM NaCl. The results revealed that *ktn1-4* showed higher salt tolerance than *35S::KTN1* (#3), with a smaller portion of leaves being discoloured and bleached. Hoever, the survival rate of *ktn1-4* and *35S::KTN1* were both lower than that of WT (Figure S1).

To test whether the transcription of *AtKTN1* changed in response to salt stress, the expression level of *AtKTN1* gene in WT seedlings after transferring to 1/2 MS containing 200 mM NaCl was also analysed by quantitative real-time PCR. The result showed that there is a slight induction of *AtKTN1* tanscript after 6 h, but then the expression level decreased significantly as the treatment time increased, which droped to about one quarter at 48 h. (Figure 1F).

Taken together, these results present an interesting phenomenon, that although the knock-out of *AtKTN1* promotes seedling survival under salt stress during the early periods, both mutant line *ktn1-4* and overexpression line *35S::KTN1* show high sensitivity to NaCl treatment ultimately.

### 2.2. Depletion and Overexpression of AtKTN1 Both Caused Abnormal Cortical Microtubule Organization

**Figure 2:**
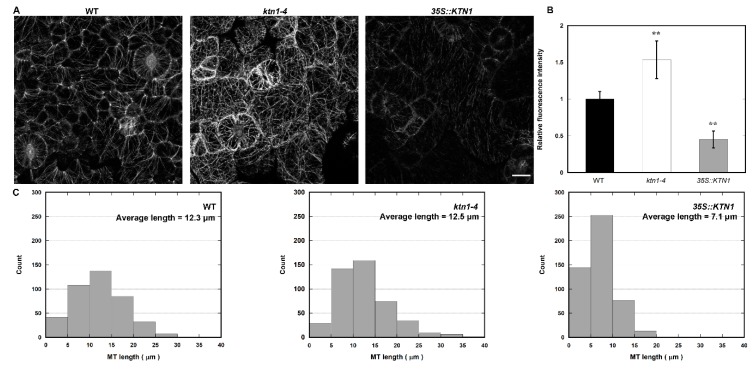
Cortical microtubule organization in cotyledon pavement cells of WT (Col-0), *ktn1-4* and *35S::KTN1* expressing GFP-TUA6. (**A**) Micrograph of microtubules in *Arabidopsis* cotyledon pavement cells of 4-d-old seedlings. Images of microtubules were acquired under the same settings. Scale bar = 10 μm. (**B**) Average fluorescence intensity of microtubules in the WT (Col-0), *ktn1-4* and *35S::KTN1*, analyzed by MBF-ImageJ software. More than 26 cells from four different seedlings were measured in each experiment. Values represent mean ± SE. ** *P* < 0.01 by a Student’s *t* test. (**C**) Histograms of microtubule length distribution were shown in WT (Col-0), *ktn1-4* and *35S::KTN1* cotyledon pavement cells. More than 400 microtubules in at least 14 cells from four seedlings were measured of each genotype were counted.

To explore whether AtKTN1 regulates salt sensitivity by affecting microtubule organization, we first observed cortical microtubules in cotyledon pavement cells of four-day-old GFP-TUA6 labeled WT, *ktn1-4* and *35S::KTN1* seedlings. It was found that microtubule organization became obviously disorganized in both *ktn1-4* and *35S::KTN1* seedlings (Figure 2A). In addition, the knock-out of *AtKTN1* led to more complex microtubule networks with higher fluorescent intensity in comparison with the WT control, whereas the *35S::KTN1* cells displayed fewer complex microtubule networks with lower fluorescent intensity (Figure 2A,B). This result indicates that the amount of microtubules was significantly enhanced in *ktn1-4* and was obviously reduced in *35S::KTN1* cells. Moreover, the microtubules appear to be more fragmented in *35S::KTN1* cells (Figure 2A,C). Thus, the data suggest that AtKTN1 promotes microtubule depolymerization, and is required for microtubule organization.

### 2.3. AtKTN1 Is Involved in Regulating Microtubule Depolymerization in Response to Salt Stress

**Figure 3:**
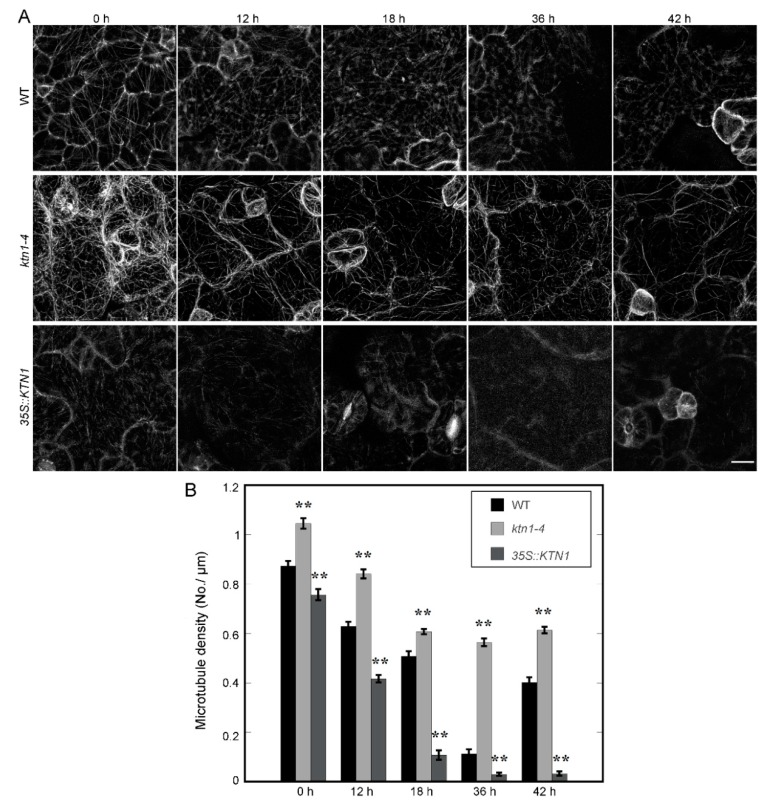
AtKTN1 modulates cortical microtubule disruption and reassembly in cotyledon pavement cells during the early stage of salt stress (0 h–42 h). (**A**) Sequential images of cortical microtubule alterations induced by 200 mM NaCl in four-day-old WT (Col-0), *ktn1-4* and *35S::KTN1* seedlings expressing GFP-TUA6 for the indicated time. Bar = 10 μm. (**B**) Quantification of cortical microtubule density in cotyledon pavement cells of WT (Col-0), *ktn1-4* and *35S::KTN1*. The data shown in (**A**) were quantified by MBF imageJ software. N ≥ 22 cells. Error bars indicate SE. ** *P* < 0.01 by a Student’s *t* test.

GFP-TUA6 labeled WT, *ktn1-4* and *35S::KTN1* seedlings were used to investigate the relationship between AtKTN1 and microtubule reorganization under salt stress. Four-day-old seedlings were transferred onto 1/2 MS supplemented with 200 mM NaCl. Microtubules in cotyledon pavement cells were imaged at indicated time, and the microtubule density were then analyzed. Before salt treatment, the microtubule density in *ktn1-4* cells (1.04 ± 0.02 No./μm) was higher than that in WT cells (0.87 ± 0.02 No./μm), and *35S::KTN1* cells displayed lower microtubule density (0.76 ± 0.02 No./μm) compared with WT. After 200 mM NaCl treatment, cortical microtubule disassembly was induced. The density of microtubule in WT, *ktn1-4* and *35S::KTN1* cells decreased to 0.63 ± 0.02 No./μm, 0.84 ± 0.02 No./μm and 0.42 ± 0.01 No./μm respectively after treatment for 12 h. Statistic analysis indicated that the microtubule density in WT cells showed significant difference with both of that in *ktn1-4* or *35S::KTN1* cells. After NaCl treatment for 18 h, microtubule disassembly was ongoing, and the density of microtubules in WT, *ktn1-4* and *35S::KTN1* cells decreased to 0.51 ± 0.02 No./μm, 0.61 ± 0.01 No./μm and 0.11 ± 0.02 No./μm respectively. Microtubules performed further depolymerization after treatment for 36 h, and the microtubule density in WT and *35S::KTN1* cells declined sharply to 0.11 ± 0.02 No./μm, 0.03 ± 0.01 No./μm. However, microtubule density in *ktn1-4* was 0.56 ± 0.02 No./μm, and the microtubule structures in cells still maintained in a comparatively organized arrays (Figure 3A,B). After extending the period of NaCl treatment to 42 h, we found that the disassembly of microtubules was rescued in WT cells. However, no recovery and reorganization of microtubules was observed in either *ktn1-4* or *35S::KTN1* cells. But the density of microtubules of *ktn1-4* cells (0.61 ± 0.01 No./μm) was still higher in comparison to that of WT (0.40 ± 0.02 No./μm) (Figure 3A,B).

**Figure 4:**
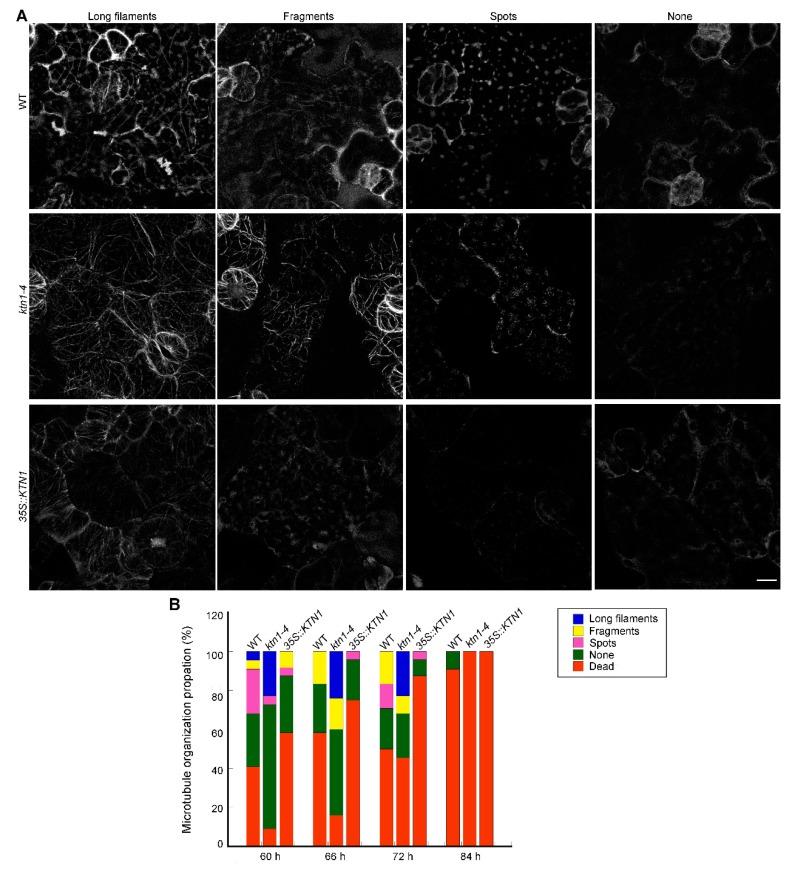
AtKTN1 regulates cortical microtubule organization in cotyledon pavement cells during the late stage of salt treatment (60–84 h). (**A**) Micrograph of different patterns of microtubule organization in cotyledon pavement cells during late stage of salt stress. Four-day-old seedlings of GFP-TUA6 labeled WT (Col-0), *ktn1-4* and *35S::KTN1* were transferred onto 1/2 MS medium supplemented with 200 mM NaCl. Different representative patterns were indicated with long filaments, fragments, spots, or nothing. Scale bar = 10 μm. (**B**) Quantification of the patterns of cortical microtubules arrays in (**A**) at indicated time. More than 11 seedlings for each line were observed at each indicated time.

In consideration of the inconsistent performances of *ktn1-4* seedlings during the whole period of NaCl treatment (Figure 1), we further extended the period of NaCl treatment and observed both of the death rates and microtubule organization in GFP-TUA6 labeled WT, *ktn1-4* and *35S::KTN1* seedlings. Different microtubule patterns were classified as long filaments, fragments, spots and none (survived but has no obvious microtubule signals) (Figure 4A). After NaCl treatment for 60 h, the death rates of WT seedlings was 41%, but those of *ktn1-4* and *35S::KTN1* were 9% and 58%, respectively. Meanwhile, the percentage of long filaments in *ktn1-4* cells (23%) was significantly higher than that of WT (5%) and *35S::KTN1* (0%). However, the frequency of none in *ktn1-4* cells (64%) was obviously higher compared with that in WT (27%) and *35S::KTN1* cells (29%) (Figure 4B). Similar trends were found at 66 h. However, after treated for 72 h, WT and *ktn1-4* seedlings suffered almost the same mortality, and the death rates were 50% and 46% respectively, while that of *35S::KTN1* seedlings was 88%. There were still a large number of long microtubules in *ktn1-4* cells (24%), while no long microtubules were found in WT and *35S::KTN1* cells. The proportion of death rates increased with the increase of treatment duration. After 84 h, no *ktn1-4* and *35S::KTN1* seedlings survived, but the survival rate of WT seedlings was 9%.

### 2.4. Defects of Dynamics in Both ktn1-4 and 35S::KTN1 Cotyledon Pavement Cells

**Figure 5:**
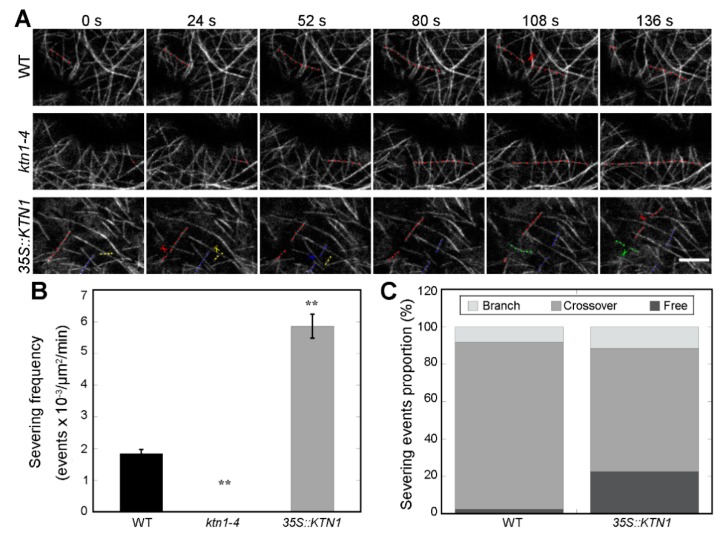
Time-lapse imaging of microtubules revealed abnormal severing frequencies in both *ktn1-4* and *35S::KTN1* cotyledon pavement cells. (**A**) Time-lapse images showing microtubule severing events in WT (Col-0), *ktn1-4* and *35S::KTN1* cells. The images presented is an optical section. A filament highlighted with colored dots underwent severing, and the corresponding colored scissors indicate the severing events. Different colors (red, blue, green and yellow) indicated different filaments. See Supplemental Movie 1-3 online for the entire series. Scale bar = 5 μm. (**B**) Quantitative comparison of severing frequencies in microtubules of WT (Col-0), *ktn1-4* and *35S::KTN1* cells (n = 6 cells for each genotype). Error bars indicate SE. ** *P* < 0.01 by a Student’s *t* test. (**C**) Quantification of the proportions of severing events (severed at branched sites, microtubule crossovers or at free microtubules) of cortical microtubules in WT (Col-0), *ktn1-4* and *35S::KTN1* cells.

Previous studies showed that Katanin p60 subunit KTN1 and the p80 subunit KTN80 perform precise microtubule severing at either microtubule branching nucleation sites or crossovers, and severing at CMT crossover sites in the *ktn1-2* mutant hypocotyl and leaf pavement cells was abolished [30,32]. To further analyze why disorganized cortical microtubule structures were found in both *ktn1-4* and *35S::KTN1* cells, the severing frequencies were investigated in four-day-old GFP-TUA6 labeled WT, *ktn1-4* and *35S::KTN1* cotyledon pavement cells by live-cell imaging experiments (Figure 5A, Movie S1–S3). As expected, the depletion of *AtKTN1* led to the absence of severing activity in *ktn1-4* cells, whereas the overexpression of *AtKTN1* resulted in enhanced severing activity significantly in *35S::KTN1* cells (Figure 5A,B). The severing frequency in WT cells was 1.84 ± 0.13 events x 10^−3^ × μm^−2^ × min^−1^ (N = 6 cells), while the frequency in *ktn1-4* and *35S::KTN1* cells was 0 (N = 6 cells) and 5.86 ± 0.38 events × 10^−3^ × μm^−2^ × min^−1^ (N = 6 cells), respectively. AtKTN1 mainly performs severing at either microtubule branching nucleation sites or crossovers [32]. In addition, among all the severing events observed, the proportions of the severing sites were also analyzed. The frequencies of severing events performed at branched nucleation site, crossovers, and free microtubules in WT cells were 8.24%, 89.41% and 2.35% respectively. While in *35S::KTN1* cells, the corresponding proportions of these sites were 11.56%, 65.90% and 22.54%, respectively (Figure 5A,C). These results indicated that the knock-out of *AtKTN1* abolished severing activity, whereas their overexpression led to a higher frequency of severing, especially at free microtubules.

**Figure 6:**
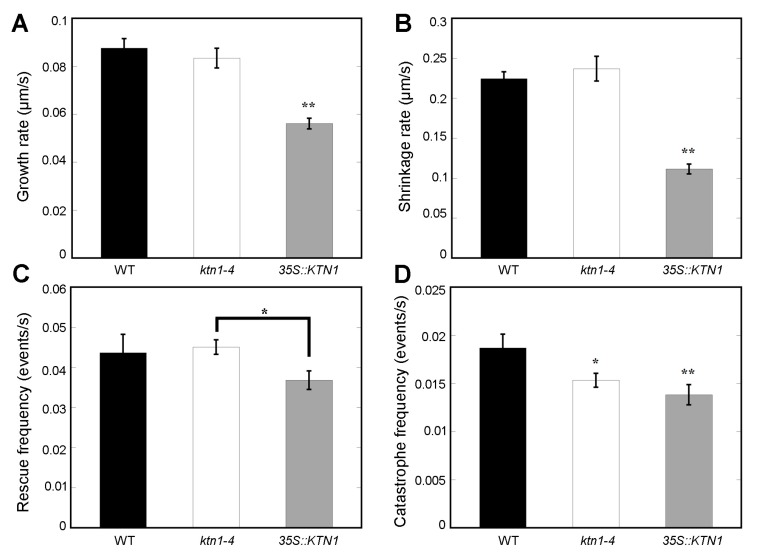
Dynamic parameters of single microtubules in cotyledon pavement cells. Quantification of microtubule growth rates (**A**), shrinkage rates (**B**), rescue frequencies (**C**) and catastrophe frequencies (**D**) in WT (Col-0), *ktn1-4* and *35S::KTN1* cells. More than 38 microtubules for each type of cells were selected to measure parameters associated with single microtubule dynamics. Error bars indicate SE. * *P* < 0.05 and ** *P* < 0.01 by a Student’s *t* test.

Taking into consideration that cortical microtubule structures have a close relation with the dynamics of individual microtubules, further inspection of microtubule dynamic parameters of cotyledon pavement cells were investigated. The result showed that microtubules in *ktn1-4* cells exhibited similar microtubule growth rates (0.083 ± 0.004 μm/s) and shrinkage rates (0.24 ± 0.015 μm/s) by comparison to that of WT (0.087 ± 0.004 μm/s and 0.22 ± 0.009 μm/s, respectively). But microtubules in *35S::KTN1* cells presented obviously lower growth rates (0.056 ± 0.002 μm/s) and shrinkage rates (0.11 ± 0.006 μm/s) (Figure 6A,B). In addition, there was no significant difference in microtubule rescue (transition from shrinkage to growth) frequency in *ktn1-4* and *35S::KTN1* cells compared with in WT cells (Figure 6C). However, the catastrophe (transition from growth to shrinkage) frequency in both of *ktn1-4* (0.015 ± 0.001 events/s) and *35S::KTN1* cells (0.014 ± 0.001 events/s) were obviously lower compared with that of WT cells (0.019 ± 0.001 events/s) (Figure 6D). The decrease of catastrophe frequency in *ktn1-4* indicated that AtKTN1 increases the frequency of transition from growth to shrinkage. Further, the decline of catastrophe frequency in *35S::KTN1* cells suggested that an excess expressional level of *AtKTN1* may also impede microtubules from growth to shrinkage. Taken together, AtKTN1 regulates microtubule organization by manipulating microtubule severing.

### 2.5. AtKTN1 Involved in Stress Related Signaling Pathways

**Figure 7:**
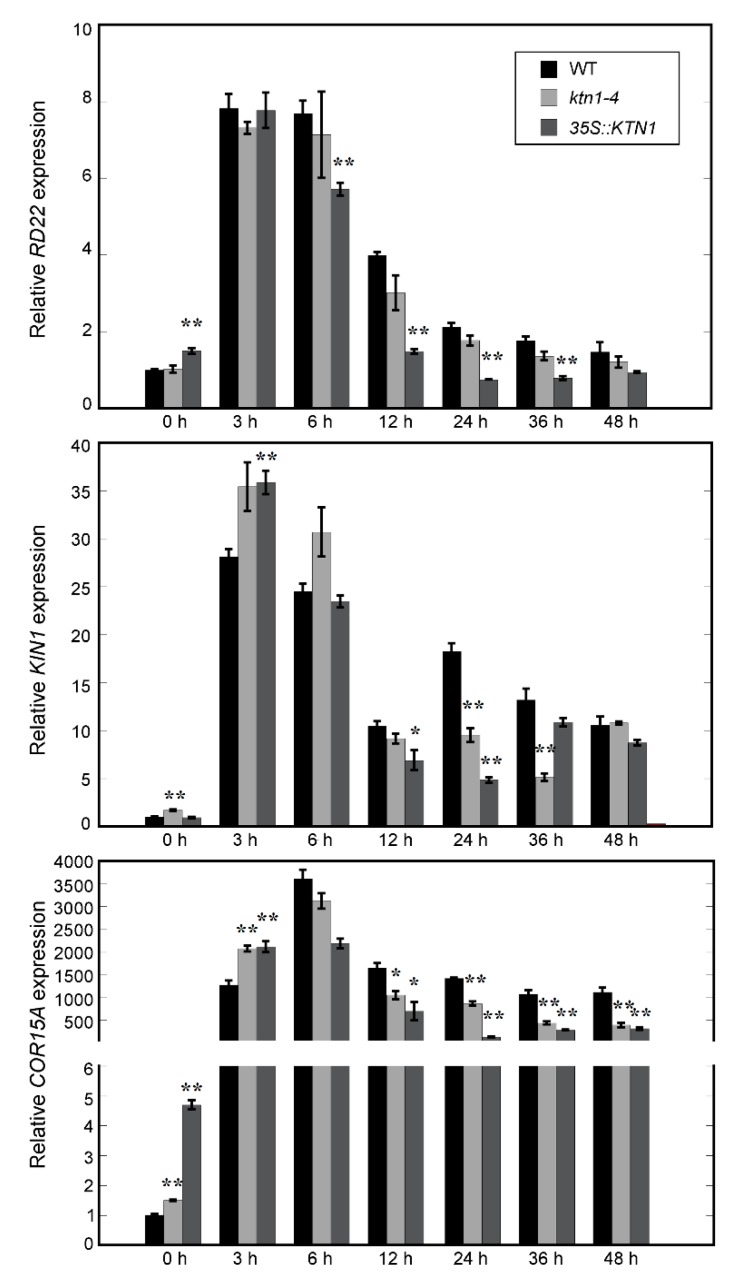
The effects of salt treatment on the transcript levels of stress-responsive genes in *Arabidopsis*. For salt treatment, four-day-old WT (Col-0), *ktn1-4* and *35S::KTN1* seedlings were transferred onto 1/2 MS with 200 mM NaCl for the indicated time period. The transcript levels of every gene in WT (0 h) was normalized as 1.0. Error bars indicate SE. * *P* < 0.05 and ** *P* < 0.01 by a Student’s *t* test.

Several stress induced genes, including *RD22*, *KIN1,* and *COR15A*, were known to confer salt stress tolerance in *Arabidopsis* [36]. To investigate whether KTN1 involved in stress related signal pathways, four-day-old WT (Col-0), *ktn1-4* and *35S::KTN1* seedlings were transferred onto 1/2 MS with 200 mM NaCl for indicated time period and the expression of these genes were quantified by real-time PCR analysis. The results showed that the expressional level of *RD22* were induced in all three genotypic lines after 3 h of treatment. However, *RD22* expression level decreased obviously faster in *35S::KTN1* than in WT and *ktn1-4*. After treatment for 12 h, the *RD22* transcript declined to the same level as the 0 h sample in *35S::KTN1*, while that of WT and *ktn1-4* were still 4- and 3-fold compared with the 0 h samples. Similar results were observed about the expression profiles of *KIN1* and *COR15A*, the WT showed a higher induction of the two genes in comparison with *ktn1-4* and *35S::KTN1* during long term salt stress (Figure 7). Downregulation of these stress induced genes in *ktn1-4* and *35S::KTN1* seedlings compared with in WT under salt stress suggested an abnormal expression level of *AtKTN1* also has a negative effect on the salt tolerance of *Arabidopsis* plants.

## 3. Discussion

KTN1 plays critical roles in regulating plant growth and development, and *AtKTN1* mutants display pleiotropic phenotypic defects with abnormal organs [22,24,30,37,38,39]. Previous studies have also suggested roles of KTN1 in response to environment stimuli, such as mechanical stress, blue light perception, and hormonal signaling [35,38,40,41]. In the present study, we demonstrated that AtKTN1 also played an important role in the salt tolerance of *Arabidopsis*.

### 3.1. AtKTN1 Mediated Salt Tolerance in Arabidopsis

Previous studies always demostrated consistent response to salt stress during the whole treatment, and few reports showed that *Arabidopsis* seedlings present variations of stress responses during different time points under salt treatment [15,16,17,19]. In this study, our results about salt tolerance analysis showed that the survial rate of *ktn1-4* was obviously higher than WT in the early stage of salt stress, and then decreased significantly as the treatment time increased. However, the overexpression of *AtKTN1* significantly decreased salt tolerance in *Arabidopsis* (Figure 1). These results proposed an interesting question concerning roles of AtKTN1 in salt tolerance, however, a similar situation wasn’t observed when four-week-old soiled grown seedlings were irratating with 400 mM NaCl, and both *ktn1-4* and *35S::KTN1* seedlings showed a lower survival rate compared with WT under stress (Figure S1).

### 3.2. AtKTN1 Is Involved in Regulating Cortical Microtubule Organization and Dynamics

Considering the crucial functions of AtKTN1 in modulating microtubule organization, and roles of microtubule cytoskeleton in plant growth, development, and also in responses to envrionmental stimuli, including abiotic and biotic stresses, we observed microtubule structures in cells of each genotype [2,7,8,10,25]. We found that the number of microtubules in *ktn1-4* was significantly improved with higher fluorescent intensity than Wt., while microtubules in *35S::KTN1* cells displayed lower fluorescent intensity with shortened fragments (Figure 2), suggesting the severing activity of AtKTN1 and its significance to maintaining organized microtubule structures. This is consistent with the previous report that the overexpression of *AtKTN1* caused numerous short microtubules in pavement cells, guard cells, and hypocotyl epidermal cells [42]. It was pointed out that microtubules first undergo disassembly followed by assembly under salt stress, and this reorganization is vital for salt resistant of plants [9,12]. The data obtained in this study showed that the overexpression of *AtKTN1* led to a sharp depolymerization but no recovery of cortical microtubules at indicated time points under salt stress, whereas the reorganization of microtubules was induced in WT cells after salt treatment for 42 h. Although the depletion of *AtKTN1* also caused obvious the depolymerization of microtubules, the density of microtubules still maintained a relatively high and steady level compared with WT cells. Further, no obvious reorganization was observed (Figure 3), implying that the loss-of-function of AtKTN1 also affected the reorganization of the microtubule array in *ktn1-4* cells. In addition, our findings about long-time salt stress analysis demonstrated that even after treatment for 60 h and 66 h, *ktn1-4* seedlings displayed a higher percentage of long filaments and lower death rate than WT (Figure 4). There results provided explanations for why *ktn1-4* seedlings showed higher survival rate than WT in early stage of salt stress. Formation of newly microtubule arrays has shown to be necessary for salt resisitant [12]. Microtubule-stabilizing protein WDL5 functions as positive modulator of microtubule reorganization under salt stress, and overexpression of WDL5 resulted in significantly higher survival rate contrasted to WT [19]. However, in the present study, we have not observed substantial signs of recovery of microtubule alignment either in early stage or in late stage in *ktn1-4* cells. Further, this disability of recovery in turn led to death earlier, consistent with the results reported previously that the supplementation of microtubule-stabilizing drugs result in dramatic salt sensitivity and reduced survial rate [9].

Genetic and live cell imaging studies demonstrated that the severing activity of KTN1 plays vital roles in driving the dynamic remodeling of cortical microtubule alignment [24,25,29,43]. Our findings showed that severing activity of AtKTN1 was lost in *ktn1-4* mutant cells (Figure 5). Previous studies have reported the failure of severing microtubules in *ktn1-2* mutant cells and the disruption of recruitment of AtKTN1 to microtubules in *ktn80.1234* quadruple mutant cells [32,34]. Our results also suggested that the overexpression of *AtKTN1* resulted in a higher frequency of severing, especially at free microtubules (Figure 5), other than branching sites and crossover sites. Analysis of microtubule dynamic parameters in each genotype of cells demonstrated that the catastrophe frequency was obviously decreased in *ktn1-4* cells (Figure 6), which could help explaining the considerable reduction of microtubule disassembly, at least partly. However, in *ktn1-2* mutant cells, both catastrophe frequency and rescue frequency were significantly affected [34]. The quantification analysis also showed a dramatic reduction of growth rate, shrinkage rate, and catastrophe frequency in *35S::KTN1* cells, suggesting a lower dynamic level. Combined with the lower amount and higher fragmentation of microtubules, these results may explain disorganized cortical microtubules and the lack of capacity for microtubule reorganization in *35S::KTN1* cells. However, more evidence concerning how the overexpression of *AtKTN1* could lead to the promotion of severing at free microtubules will need be to answered.

### 3.3. AtKTN1 Is Involved in Stress Related Signaling Pathways

A growing number of recent studies suggest that microtubule cytoskeleton can be a target of various hormonal and stress signaling pathways in plants [2,7,10,25,44]. Our data showed that the expression levels of these stress-related genes decrease rapidly at the late stage of salt stress in *ktn1-4* and *35S::KTN1* (Figure 7), suggesting that AtKTN1 is also involved in stress-related signaling pathways. In addition, this fact also supports the results that both the knock-out and overexpression of *AtKTN1* led to lower survival rates at the late stage of salt stress in *Arabidopsis*.

## 4. Materials and Methods

### 4.1. Plasmid Constructions

For the construction of *35S::AtKTN1*, the *AtKTN1* (At1g80350) genomic region were subcloned with the primers *AtKTN1* genomic For/*AtKTN1* genomic Rev (Table S1) from genomic DNA. The resulting PCR product was cloned into pEASY-blunt vector, and subsequently inserted into the binary vector pCambia 1301-*35S::NOS* between the SalI and EcoRI sites. The *35S::AtKTN1* fusion construct was introduced into *Agrobacterium tumefaciens* strain GV3101 and used to transform WT (Col-0) plants by floral dip method [45]. Transgenic lines were selected on Murashige and Skoog culture medium containing hygromycin (25 μg/mL) and the transcript level of *AtKTN1* were identified by RT-PCR.

### 4.2. Plant Material and Growth Conditions

The ecotype Columbia-0 (Col-0) of *Arabidopsis thaliana* was used as the WT plant in the experiment. Other plant materials are listed as follows: *ktn1-4* (Sail_551_D06), and two *AtKATANIN1* overexpressing lines, *35S::AtKTN1* (#3,#4) and *35S::GFP*-*TUA6* [46]. The *ktn1-4* (Sail_551_D06) mutant and *35S::GFP*-*TUA6* line were kind gifts from Prof. Shanjin Huang (Tsinghua University, Beijing, China). Further, *ktn1-4* and *35S::AtKTN1* lines expressing GFP-TUA6 were obtained by cross each line with *35S::GFP*-*TUA6* transgenic line respectively.

The plants were cultured on half Murashige and Skoog medium supplemented with 1% sucrose (*w/v*) and 0.85% agar (*w/v*), incubated at 4 °C for 2 days, and then transferred to a culturing room with dark/light cycles of 8/16 h for 1 week. Positive seedlings were transferred and cultured on soil in culture room.

### 4.3. Salt Sensitivity Assay

For phenotypic analysis on plate, four-day-old seedlings of wild type, *ktn1-4* and *35S::AtKTN1* (#3, #4) were transplanted to plates without or supplemented with 150 mM and 200 mM sodium chloride (NaCl). After 2 days, plants were photographed and the survival rate was calculated every 12 h till 6-day treatments. At least 36 seedlings were observed for each line, and each experiment was repeated three times.

### 4.4. RT-PCR and Quantitative Real-Time PCR Analysis

For RT-PCR analysis, to assess the expression level of the gene *AtKTN1*, total RNA was isolated from seven-day seedlings of wild type, *ktn1-4* and *35S::AtKTN1* (#3, #4) with the Trizol reagent (Invitrogen, Carlsbad, CA, USA) according to the manufacturer’s instructions. For cDNA synthesis, 2 μg of total RNA from different samples was used for reverse transcription with Revert Aid First Strand cDNA Synthesis Kit (Thermo Scientific, Waltham, MA, USA) according to the manufacturer’s recommendations. The expression level of the full-length of *AtKTN1* was determined with the primer pair *AtKTN1* genomic For/*AtKTN1* genomic Rev (Table S1), the gene eIF4A was used as the endogenous control. To analyze the transcript levels of stress-related genes in WT, *ktn1-4,* and *35S::KTN1* (#3) seedlings under salt stress, four-day-old seedlings were transferred onto 1/2 MS with 200 mM NaCl for an indicated time period. Total RNA extraction and cDNA synthesis was performed as before. Quantitative real-time PCR was performed with Roche LightCyler 96^®^ in a 20 μL reaction volume containing SYBR Green dye (FastStart Essential DNA Green Master, Roche, Mannheim, Germany). Further, *eIF4A* was chosen as an internal control. Relative expression levels were estimated using the 2^−∆∆*C*t^ method [47]. All primers used in the study are listed in Supplementary Table S1.

### 4.5. Visualization of Cortical Microtubules in Cotyledon Pavement Cells by Confocal Microscopy

For cortical microtubule analysis, epidermal pavement cells of four-day-old WT, *ktn1-4* and *35S::AtKTN1* (#3) seedlings expressing GFP-TUA6 were observed with Leica TCS SP8 laser scanning confocal microscope, with excitation of 488 nm argon laser, and emission wave length range of 505–525 nm. The projection of z-stack images was performed with Image J (http://rsbweb.nih.gov/ij/, version 1.47g).

To quantify the cortical microtubules length, more than 400 microtubules in at least 14 cells from 4 seedlings were measured in each experiment, repeated at least three times. To compare the fluorescent intensity of GFP in pavement cells between WT, *ktn1-4* and *35S::AtKTN1* (#3) seedlings, all optical sections were acquired under identical conditions. Quantification of the fluorescent intensity was performed by measuring the mean gray value using Image J software. For each line, more than 26 cells from 4 different seedlings were measured in each experiment, repeated at least three times. To quantify the individual microtubule dynamics, epidermal pavement cells of four-day-old seedlings also were observed under a Leica TCS SP8 microscope. The time interval for time-lapse images was 4 s during the course of 400 s. Parameters such as growth and shrinkage rates, rescue and catastrophe frequencies were calculated according to the published methods [48,49]. Only MTs that could be traced over two successive frames were selected for measuring the parameters associated with single MT dynamics. For each line, more than 20 single microtubules from 3 different seedlings were measured in each experiment, repeated at least three times. Severing frequency in wild type, *ktn1-4* and *35S::AtKTN1* (#3) cells were measured from time-lapse movies and were expressed as events × 10^−3^/μm^2^/min [34].

### 4.6. Measurement of Microtubule Density and Quantification of Different Extent of Disassembled Cortical Microtubule Structures in Cotyledon Pavement Cells Under Salt Treatment

To analyze the impact of KTN1 on microtubule organization changes in pavement cells during salt treatment, 4-day-old WT, *ktn1-4,* and *35S::AtKTN1* (#3) seedlings expressing GFP-TUA6 were transferred to plates supplemented with 200 mM NaCl. After another 12–42 h after treatment, images of cortical microtubule in pavement cells from control and salt treated samples were captured as demonstrated before. The density of microtubules (microtubule number per unit distance) was quantified [10,12,16,50]. For each line, more than 22 cells from 4 different seedlings were measured in each experiment, repeated at least three times. Considering that 2 days of 200 mM salt treatment initiates death in seedlings, we quantified death rates and different extents of disassembled cortical microtubule structures of cotyledon pavement cells after 60–84 h salt treatment, to further analyse the influence of KTN1 on microtubule organization.

### 4.7. Statistical Analysis

Statistical significance analyses were performed in KaleidaGraph 4.03 (Synergy Software, Reading, PA, USA).

## 5. Conclusions

Taken together, our results highlight the roles of AtKTN1 in response to salt stress by modulating microtubule organization, microtubule disruption and recovery, and its involvement in stress related signaling pathways.

## Supplementary Materials

Supplementary materials can be found at https://www.mdpi.com/1422-0067/21/1/138/s1.

## Abbreviations

AtKTN1	ATKATANIN1
MAP65-1	Microtubule-Associated Protein 65-1
ROPs	Rho-related GTPase from plants
H2Bub1	Histone H2B monoubiquitination
OsMAR1	*Oryza sativa* E3 ligase Microtubule-Associated RING finger protein 1
OCPI2	*Oryza sativa* chymotrypsin protease inhibitor 2
WDL5	WAVEDAMPENED2-LIKE5
MAPs	Microtubule Associated Proteins

## References

[1] Munns R., Tester M. (2008). Mechanisms of salinity tolerance. Annu. Rev. Plant Biol..

[2] Yang Y., Guo Y. (2018). Elucidating the molecular mechanisms mediating plant salt-stress responses. New Phytol..

[3] Yang Y., Guo Y. (2018). Unraveling salt stress signaling in plants. J. Integr. Plant Biol..

[4] Zhu J.K. (2002). Salt and drought stress signal transduction in plants. Annu. Rev. Plant Biol..

[5] Cyr R.J. (1994). Microtubules in Plant Morphogenesis: Role of the Cortical Array. Annu. Rev. Cell Biol..

[6] Nick P. (1998). Signals, motors, morphogenesis the cytoskeleton in plant development. Plant Biol..

[7] Wasteneys G.O. (2004). Progress in understanding the role of microtubules in plant cells. Curr. Opin. Plant Biol..

[8] Lloyd C. (2011). Dynamic Microtubules and the Texture of Plant Cell Walls. Int. Rev. Cell Mol. Biol..

[9] Wang C., Li J., Yuan M. (2007). Salt tolerance requires cortical microtubule reorganization in *Arabidopsis*. Plant Cell Physiol..

[10] Fujita S., Pytela J., Hotta T., Kato T., Hamada T., Akamatsu R., Ishida Y., Kutsuna N., Hasezawa S., Nomura Y. (2013). An atypical tubulin kinase mediates stress-induced microtubule depolymerization in *Arabidopsis*. Curr. Biol..

[11] Shoji T., Suzuki K., Abe T., Kaneko Y., Shi H., Zhu J.K., Rus A., Hasegawa P.M., Hashimoto T. (2006). Salt stress affects cortical microtubule organization and helical growth in *Arabidopsis*. Plant Cell Physiol..

[12] Wang S., Kurepa J., Hashimoto T., Smalle J.A. (2011). Salt stress-induced disassembly of *Arabidopsis* cortical microtubule arrays involves 26S proteasome-dependent degradation of SPIRAL1. Plant Cell.

[13] Balancaflor E.B., Hasenstein K.H. (1995). Growth and microtubule orientation of *Zea mays* roots subjected to osmotic stress. Int. J. Plant Sci..

[14] Dhonukshe P., Laxalt A.M., Goedhart J., Gadella T.W., Munnik T. (2003). Phospholipase D activation correlates with microtubule reorganisation in living plant cells. Plant Cell.

[15] Zhang Q., Lin F., Mao T., Nie J., Yan M., Yuan M., Zhang W. (2012). Phosphatidic acid regulates microtubule organisation by interacting with MAP65-1 in response to salt stress in *Arabidopsis*. Plant Cell.

[16] Li C., Lu H., Li W., Yuan M., Fu Y. (2017). A ROP2-RIC1 pathway fine-tunes microtubule reorganization for salt tolerance in *Arabidopsis*. Plant Cell Environ..

[17] Zhou S., Chen Q., Sun Y., Li Y. (2017). Histone H2B monoubiquitination regulates salt stress-induced microtubule depolymerization in *Arabidopsis*. Plant Cell Environ..

[18] Park Y.C., Chapagain S., Jang C.S. (2018). The microtubule-associated RING finger protein 1 (OsMAR1) acts as a negative regulator for salt-stress response through the regulation of OCPI2 (*O. sativa* chymotrypsin protease inhibitor 2). Planta.

[19] Dou L., He K.K., Higaki T., Wang X., Mao T. (2018). Ethylene signaling modulates cortical microtubule reassembly in response to salt stress. Plant Physiol..

[20] Dixit R., Cyr R. (2004). The cortical microtubule array: From dynamics to organization. Plant Cell.

[21] Elliott A., Shaw S.L. (2018). Update: Plant cortical microtubule arrays. Plant Physiol..

[22] Bichet A., Desnos T., Turner S., Grandjean O., Hofte H. (2001). Botero1 is required for normal orientation of cortical microtubules and anisotropic cell expansion in *Arabidopsis*. Plant J..

[23] Stoppin-Mellet V., Gaillard J., Vantard M. (2002). Functional evidence for in vitro microtubule severing by the plant katanin homologue. Biochem J..

[24] Bouquin T., Mattsson O., Naested H., Foster R., Mundy J. (2003). The *Arabidopsis lue1* mutant defines a katanin p60 ortholog involved in hormonal control of microtubule orientation during cell growth. J. Cell Sci..

[25] Nakamura M., Ehrhardt D.W., Hashimoto T. (2010). Microtubule and katanin dependent dynamics of microtubule nucleation complexes in the acentrosomal *Arabidopsis* cortical array. Nat. Cell Biol..

[26] McNally F.J., Vale R.D. (1993). Identification of katanin, an ATPase that severs and disassembles stable microtubules. Cell.

[27] Roll-Mecak K., McNally F.J. (2010). Microtubule-severing enzymes. Curr. Opin. Cell Biol..

[28] Hartman J.J., Mahr J., McNally K., Okawa K., Iwamatsu A., Thomas S., Cheesman S., Heuser J., Vale R.D., McNally F.J. (1998). Katanin, a microtubule-severing protein, is a novel AAA ATPase that targets to the centrosome using a WD40-containing subunit. Cell.

[29] Wightman R., Chomicki G., Kumar M., Carr P., Turner S.R. (2013). SPIRAL2 determines plant microtubule organization by modulating microtubule severing. Curr. Biol..

[30] Zhang Q., Fishel E., Bertroche T., Dixit R. (2013). Microtubule severing at crossover sites by katanin generates ordered cortical microtubule arrays in *Arabidopsis*. Curr. Biol..

[31] Wang G., Wang C., Liu W., Ma Y., Dong L., Tian J., Yu Y., Kong Z. (2018). Augmin antagonizes katanin at microtubule crossovers to control the dynamic organization of plant cortical arrays. Curr. Biol..

[32] Wang C., Liu W., Wang G., Li J., Dong L., Han L., Wang Q., Tian J., Yu Y., Gao C. (2017). KTN80 confers precision to microtubule severing by specific targeting of katanin complexes in plant cells. EMBO J..

[33] Komorisono M., Ueguchi-Tanaka M., Aichi I., Hasegawa Y., Ashikari M., Kitano H., Matsuoka M., Sazuka T. (2005). Analysis of the *rice* mutant *dwarf and gladius leaf 1*. Aberrant katanin-mediated microtubule organization causes up-regulation of gibberellin biosynthetic genes independently of gibberellin signaling. Plant Physiol..

[34] Komis G., Luptovčiak I., Ovečka M., Samakovli D., Šamajová O., Šamaj J. (2017). Katanin effects on dynamics of cortical microtubules and mitotic arrays in *Arabidopsis thaliana* revealed by advanced live-cell imaging. Front. Plant Sci..

[35] Lin D., Cao L., Zhou Z., Zhu L., Ehrhardt D., Yang Z., Fu Y. (2013). Rho GTPase signaling activates microtubule severing to promote microtubule ordering in *Arabidopsis*. Curr. Biol..

[36] Shi H., Qian Y., Tan D.X., Reiter R.J., He C. (2015). Melatonin induces the transcripts of *CBF/DREB1s* and their involvement in both abiotic and biotic stresses in *Arabidopsis*. J. Pineal. Res..

[37] Webb M., Jouannic S., Foreman J., Linstead P., Dolan L. (2002). Cell specification in the *Arabidopsis* root epidermis requires the activity of *ECTOPIC ROOT HAIR* 3-a katanin-p60 protein. Development.

[38] Lindeboom J.J., Nakamura M., Hibbel A., Shundyak K., Gutierrez R., Ketelaar T., Emons A.M., Mulder B.M., Kirik V., Ehrhardt D.W. (2013). A mechanism for reorientation of cortical microtubule arrays driven by microtubule severing. Science.

[39] Luptovčiak I., Samakovli D., Komis G., Šamaj J. (2017). KATANIN 1 is essential for embryogenesis and seed formation in *Arabidopsis*. Front. Plant Sci..

[40] Uyttewaal M., Burian A., Alim K., Landrein B., Borowska-Wykręt D., Dedieu A., Peaucelle A., Ludynia M., Traas J., Boudaoud A. (2012). Mechanical stress acts via katanin to amplify differences in growth rate between adjacent cells in *Arabidopsis*. Cell.

[41] Sampathkumar A., Krupinski P., Wightman R., Milani P., Berquand A., Boudaoud A., Hamant O., Jönsson H., Meyerowitz E.M. (2014). Subcellular and supracellular mechanical stress prescribes cytoskeleton behavior in *Arabidopsis* cotyledon pavement cells. Elife.

[42] Stoppin-Mellet V., Gaillard J., Vantard M. (2006). Katanin’s severing activity favors bundling of cortical microtubules in plants. Plant J..

[43] Wightman R., Turner S.R. (2007). Severing at sites of microtubule crossover contributes to microtubule alignment in cortical arrays. Plant J..

[44] Zhu Y., Zuo M., Liang Y., Jiang M., Zhang J., Scheller H.V., Tan M., Zhang A. (2013). MAP65-1a positively regulates H_2_O_2_ amplification and enhances brassinosteroid-induced antioxidant defence in *maize*. J. Exp. Bot..

[45] Clough S.J., Bent A.F. (1998). Floral dip: A simplified method for Agrobacterium-mediated transformation of *Arabidopsis thaliana*. Plant J..

[46] Lu L., Lee Y.R., Pan R., Maloof J.N., Liu B. (2005). An internal motor kinesin is associated with the golgi apparatus and plays a role in trichome morphogenesis in *Arabidopsis*. Mol. Biol. Cell.

[47] Livak K.J., Schmittgen T.D. (2001). Analysis of relative gene expression data using real-time quantitative PCR and the 2(-Delta Delta C(T)) Method. Methods.

[48] Kawamura E., Wasteneys G.O. (2008). MOR1, the *Arabidopsis thaliana* homologue of *Xenopus* MAP215, promotes rapid growth and shrinkage, and suppresses the pausing of microtubules in vivo. J. Cell Sci..

[49] Yao M., Wakamatsu Y., Itoh T.J., Shoji T., Hashimoto T. (2008). *Arabidopsis* SPIRAL2 promotes uninterrupted microtubule growth by suppressing the pause state of microtubule dynamics. J. Cell Sci..

[50] Ishida T., Thitamadee S., Hashimoto T. (2007). Twisted growth and organisation of cortical microtubules. J. Plant Res..

