# Supplementary material for: AtKATANIN1 Modulates Microtubule Depolymerization and Reorganization in Response to Salt Stress in Arabidopsis
Source: Int J Mol Sci. 2019 Dec 24;21(1):138. doi: 10.3390/ijms21010138 (PMC6981882; doi:10.3390/ijms21010138)
Supplement: Supplementary file 1 [file ijms-21-00138-s001.zip › ijms-657724 supplementary done/supplemental/wqn supplemental material revised.pdf]

AtKATANIN1 modulates microtubule depolymerization and reorganization in response to salt stress in *Arabidopsis*

Jie Yang[1], Bang An[1], Hongli Luo[1], Chaozu He[1], Qiannan Wang[1]*

[1]Hainan Key Laboratory for Sustainable Utilization of Tropical Bioresource, College of Tropical Crops, Hainan University, Haikou 570228, China

Supplemental material

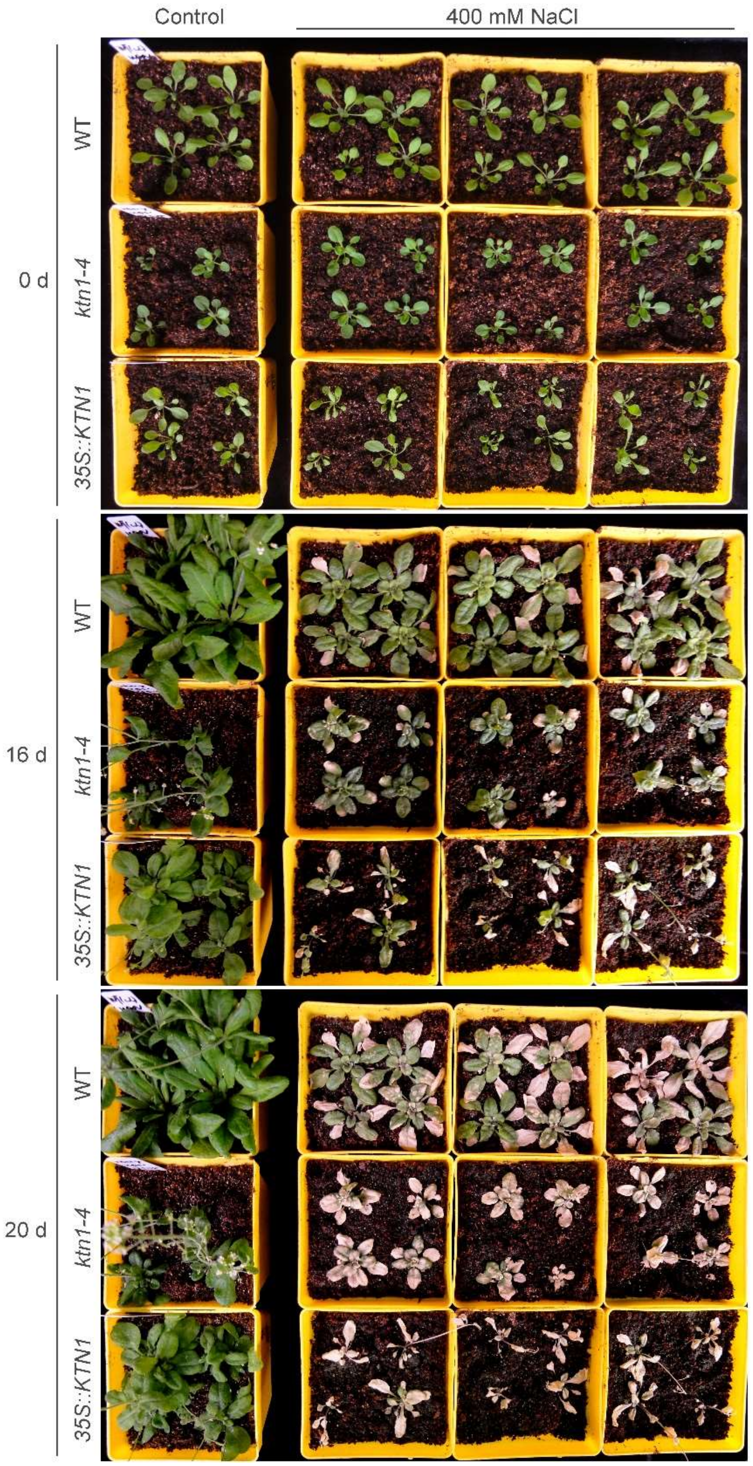

Figure S1. Both knock-out and overexpression of *AtKTN1* caused lower salt tolerance of seedlings in soil.

Four-week-old seedlings grown in soil were flood irrigated with water or 400 mM NaCl every 4 days. Images were taken at 16 d and 20 d after salt treatment respectively.

Table S1. List of the primers used in this study.

**Primer name**	**Sequence (5' to 3')**
AtKTN1 genomic For	GTCGACATGGTGGGAAGTAGTAAT
AtKTN1 genomic Rev	GAATTCTTAAGCAGATCCAAACTC
eIF4A For	CAGAGAACACTCCAACCTGAATC
eIF4A Rev	GGGTATCTATGCTTACGGTTTCG
QRT AtKTN1 For	CCCTGTTGGCAAAAGCTGTT
QRT AtKTN1 Rev	GCATATGCCCTAGCCAGATCA
QRT AtRD22 For	CCCATTCGCGGTGTTCTACT
QRT At RD22 Rev	CCAAGTGGTTTGGGTTCCAA
QRT AtKIN1 For	GCTGGCAAAGCTGAGGAGAA
QRT AtKIN1 Rev	CCGGTCTTGTCCTTCACGAA
QRT AtCOR15A For	GTGACGGCAACATCCTCGAT
QRT AtCOR15A Rev	CCCAATGTATCTGCGGTTTCA
QRT eIF4A For	CGTAAGCATAGATACCCCTAAGAA
QRT eIF4A Rev	TGACCAGAGGCTGAATGAAGT

Movie S1.-S3. Time-lapse movies of microtubule severing in cotyledon pavement cells of GFP-TUA6 labeled WT (Movie S1.), *ktn1-4* (Movie S2.) and *35S::KTN1* (Movie S3.) seedlings corresponds to the diagram shown in Figure 3A.

Images were collected at 4-s intervals and compressed into a movie with a display rate of 5 frames per second. The growing end of microtubule of interest is indicated by a yellow arrowhead, while the severing site and the broken end is marked by a red arrowhead. Scale bars= 5 μm.
