# Supplementary figures and images for: AtKATANIN1 Modulates Microtubule Depolymerization and Reorganization in Response to Salt Stress in Arabidopsis
Source: Int J Mol Sci. 2019 Dec 24;21(1):138. doi: 10.3390/ijms21010138 (PMC6981882; doi:10.3390/ijms21010138)

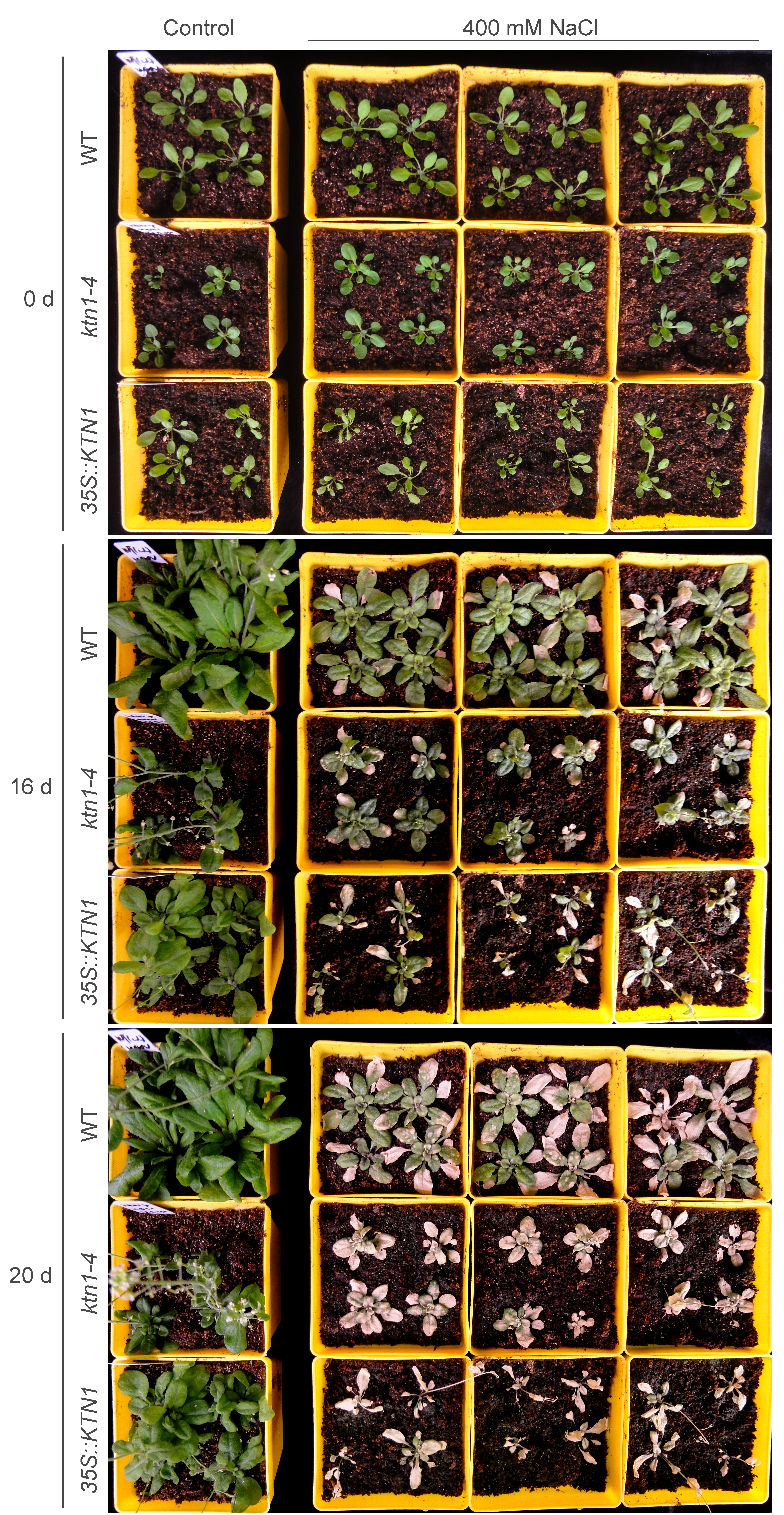

Supplement: Supplementary file 1 [file ijms-21-00138-s001.zip › ijms-657724 supplementary done/supplemental/Fig S1.tif]
